# Different Hatching Rates of Floodwater Mosquitoes *Aedes sticticus*, *Aedes rossicus* and *Aedes cinereus* from Different Flooded Environments

**DOI:** 10.3390/insects12040279

**Published:** 2021-03-25

**Authors:** Anders Lindström, Disa Eklöf, Tobias Lilja

**Affiliations:** Department of Microbiology, National Veterinary Institute, 75189 Uppsala, Sweden; anders.lindstrom@sva.se (A.L.); disa.eklof@sva.se (D.E.)

**Keywords:** floodwater mosquitoes, *Aedes sticticus*, *Aedes rossicus*, *Aedes cinereus*, egg laying, oviposition

## Abstract

**Simple Summary:**

In the lower Dalälven region, floodwater mosquitoes cause recurring problems during floods. Since 2002, flooded areas have been treated with *Bacillus thuringiensis israelensis* (Bti) to control the number of hatching floodwater mosquitoes. The area is composed of both open meadows that are grazed or mowed, as well as unmanaged open meadows and forest areas that are regularly flooded. This study compared the number of mosquito larvae that hatched from soil samples collected from these environmental categories to assess whether management of flooded areas affected mosquito abundance. The results show that fewer mosquitoes hatched from mowed meadows than from the other categories. It was further clear that *Aedes cinereus* were more abundant in open areas with tussocks while *Aedes sticticus* were dominating in forests and in open unkept areas, shrubs, and bushes. This difference is important for nuisance management since *Aedes cinereus* generally fly only short distances and mainly stay in the area where they hatched, while *Aedes sticticus* is capable to fly long distances and can cause nuisance problems in larger areas.

**Abstract:**

In the lower Dalälven region, floodwater mosquitoes cause recurring problems. The main nuisance species is *Aedes (Ochlerotatus) sticticus*, but large numbers of *Aedes (Aedes) rossicus* and *Aedes (Aedes) cinereus* also hatch during flooding events. To increase understanding of which environments in the area give rise to mosquito nuisance, soil samples were taken from 20 locations from four environmental categories: grazed meadows, mowed meadows, unkept open grassland areas and forest areas. In each location 20 soil samples were taken, 10 from random locations and 10 from moisture retaining structures, such as tussocks, shrubs, piles of leaves, logs, and roots. The soil samples were soaked with tap water in the lab, and mosquito larvae were collected and allowed to develop to adult mosquitoes for species identification. Fewer larvae hatched from mowed areas and more larvae hatched from moisture retaining structure samples than random samples. The results showed that *Aedes cinereus* mostly hatch from grazed and unkept areas and hatched as much from random samples as from structures, whereas *Aedes sticticus* and *Aedes rossicus* hatched from open unkept and forest areas and hatch significantly more from structure samples. When the moisture retaining structures in open unkept areas where *Aedes sticticus* hatched were identified it was clear that they hatched predominantly from willow shrubs that offered shade. The results suggest that *Ae. sticticus* and *Ae. cinereus* favor different flooded environments for oviposition.

## 1. Introduction

The lower Dalälven area in central Sweden is a flat area prone to flooding along around 80 km of the Dalälven River. As many flooded areas, it has recurring problems with large outbreaks of floodwater mosquitoes, mainly *Aedes sticticus*, in association with floods during spring and summer [1,2,3]. While the area is not associated with any mosquito borne disease outbreaks the mass occurrence of floodwater mosquitoes makes any outdoor activities impossible. The area has been treated with *Bacillus thuringiensis israelensis*, Bti, during floods since 2002 to control mosquito nuisance [4].

Historically, large parts of the meadows along the river were used for grazing or mowed for hay. Despite local projects, large areas are now unkept, overgrown and forested with birch (*Betula* sp.), willow (*Salix* sp.), and alder (*Alnus* sp.). Meadows are kept open as benefits for the cultural landscape and biological diversity [5]. Previous studies suggest that management of vegetation can affect mosquito abundance [6,7,8]. One previous study has seen some effect of mowing and grazing on amounts of mosquito larvae in the Dalälven area [9]. They found a 70% reduction in mosquito larvae during flooding in mowed or grazed areas compared to open but unkept areas.

Mosquitoes have been surveyed in the lower Dalälven region since 2001 [10] and the dominating mosquito species is *Ae. sticticus*, comprising 65% of collected mosquitoes, *Aedes cinereus*, 8.6%, *Aedes communis* 8.3% and *Aedes rossicus* 6.8%. Of these mosquito species, *Ae. communis* does not favor flooded areas, but lay eggs in forest areas in rain or snowmelt puddles formed during spring. *Ae. sticticus* eggs only hatch after winter conditioning and temperature after winter conditioning seem to affect hatching [11]. *Ae. sticticus* can fly up to 10 km and cause nuisance over large areas [12,13]. *Ae. cinereus* is a common, more sedentary mosquito in many areas, and seems to prefer open areas to denser bushwood [14,15]. It is multivoltine and also benefit from flooded areas [16].

Floodwater mosquitoes lay their eggs on ground that is frequently flooded, the soil should be “moist but not glistening” [17] and plant debris is beneficial. The type of vegetation influence egg laying, and *Aedes vexans* prefer dense vegetation, especially reeds such as *Phragmites australis* [18]. The structure of vegetation also seems to have an impact on egg laying [19] where debris and litter that cover the ground maintain moisture and increase survival of the eggs. In unkept open areas, the ground is often covered by withered and decaying sedge (*Carex*) forming a thick layer protecting the ground, providing good egg laying possibilities. Further, unkept areas are slowly overgrown by willows forming shrubs that provide shade and a protective layer of leaves. When an area is grazed or mowed, the ground cover is removed and moisture retaining structures (MRS), debris, and litter diminish [20]. Debris and litter cover the ground also in flooded forest areas and MRS, such as logs, piles of twigs, and leaves or roots has been shown to be preferred by mosquitoes in other parts of the world [21]. Studies around the Columbia River in North America showed that *Ae. sticticus* and *Ae. vexans* preferred wooded areas with MRS to open areas and that clearing of bush wood lowered mosquito abundance [19]. In the lower Dalälven region, a study during 2000–2002 suggested that *Ae. sticticus* was more associated to flooded Alder swamps than to flooded meadows [1]. In a regularly flooded area in Poland, egg density was correlated to environmental factors, showing that high grasses, such as *Phalaris arundinacea*, was associated with higher numbers of mosquito eggs [22].

A previous study and our unpublished observations suggested that the timing of flooding affects the number of hatched larvae where more larvae would hatch during spring and early summer compared to later in the summer [23], but no controlled experiments were performed to address this.

In this study, we wanted to study if flood water mosquitoes in the lower Dalälven area are associated with specific environmental categories, such as grazed or mowed meadows, open unkept areas or forest areas. We further wanted to study whether more mosquito eggs were associated with MRS, such as tussocks, shrubs, piles of leaves, logs, and roots than in random soil samples. We also wanted to address how season might affect hatching of mosquito eggs.

## 2. Materials and Methods

**Figure 1:**
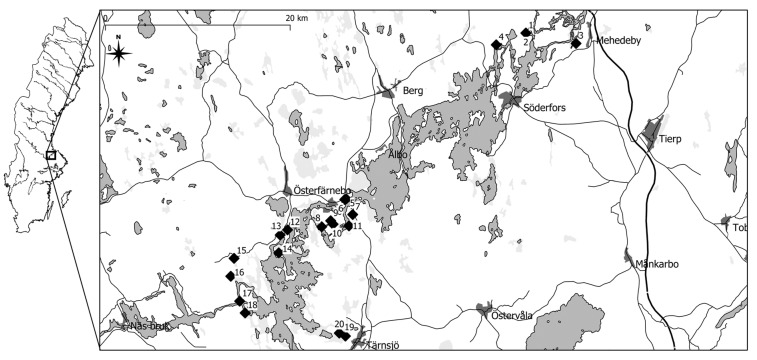
Map of the Lower Dalälven area with sample locations marked with diamonds 1–20. List of all locations in Table S2.

Soil samples were taken from twenty locations representing four environmental categories in the lower Dalälven area in central Sweden, Figure 1 and Table S2, to evaluate the effect of different environments on the number of hatched mosquito larvae. The four evaluated environmental categories were: cattle grazed areas, mowed areas, unkept open grassland areas and forest areas, all located close to the river and regularly flooded. Five areas from each category were sampled and from each area 10 samples were collected randomly in a transect through the area. Another 10 samples were actively selected and collected in each area from plant structures that we assessed could retain moisture and were assumed to be preferred by egg-laying mosquitoes. Each soil sample was 15 × 15 cm^2^ with a depth of 10 cm. Samples were collected during the spring of 2020, from 10 March to 5 May. Another set of samples from twelve locations, three from each category, were sampled during late summer from 18 August to 7 September for comparison between early and late season. There was no flood events between the spring and late summer samplings. Sample locations were recorded with photo and GPS coordinates. Each sampled MRS was also documented with a written description of the structure and surrounding vegetation. In total 640 samples were collected, 400 during spring and another 240 samples during late summer. To assess that the areas compared were flooded to a similar extent, the Grid2+ elevation data from the national land survey of Sweden, “Lantmäteriet” was used to estimate the elevation above the river of each location. The Grid2+ data plots the elevation of each 2 m square to a precision of 0.1 m and showed that the chosen locations are comparable.

The soil samples were brought to room temperature in the laboratory and placed in boxes with a depth of 19 cm. The boxes were filled with tap water so that the samples were soaked to induce hatching of mosquito eggs [24]. Forty-eight hours after soaking, larvae were picked out with pipette, counted, and placed in rearing bottles in a mix of tap water and water from the soil sample. After another 72 h (5 days after soaking) the water was poured from the sample and remaining larvae were collected and counted. Mosquito larvae were fed commercially available fish food (Sera Vipan Nature™ main feed, and Interpet Liquifry™ No2) and raised to adult mosquitoes to facilitate identification by morphology. Adult mosquitoes were killed by freezing and morphologically identified using the keys in Becker et al. [18] and Stojanovich and Scott [25].

To describe the physical differences between areas and categories, several parameters were measured. A rising-plate meter, a quadratic plywood plate measuring 25 × 25 cm^2^ with a hole in the middle sliding on a cm graded rod, was used to measure vegetation height [26]. When the end of the rod is placed at the ground, the plate is supported by surrounding soil and vegetation and the difference between the tip of the rod and the plate can be measured. For each area, the rising-plate meter was used 30 times at random points in a transect through the area, and a mean plate-meter height was calculated. Since the vegetation affects the readout, all rising-plate measurements of the mowed areas was taken before the yearly mowing. To further describe the areas a one square-meter frame was used. It was randomly placed in ten positions in each area and several measurements were taken. The distance between the frame and the ground was measured in two places on each side of the frame. This gives a measurement of the roughness of the ground similar to the rising-plate meter, but on a slightly larger scale. In addition, any MRS present in the square were measured and described, which made it possible to determine what proportion of the area was covered in structures.

The statistical analysis of the soil sample data was performed by Statisticon (Statisticon AB, Uppsala). A Mann–Whitney U-test [27] was used to analyze differences between samples taken during spring and samples taken during late summer, both in total as well as divided into random samples and MRS samples. This was also used to analyze differences between random samples and MRS samples, both divided in spring and late-summer samples as well as combined. To test differences between environmental categories, Kruskal–Wallis test [28] was used to establish whether there were any differences followed by post-hoc analysis with Dunn’s test to determine which categories differed from each other. The *p*-values were corrected for multiple tests with the Benjamini–Hochberg method. The same analysis was also used on data for each of the three most common mosquito species.

## 3. Results

In total, 7205 mosquito larvae hatched. There was a large variation in the amount of mosquito larvae hatched from each soil sample, Table S1. In slightly less than half of the samples (198 of 400) during the spring, no larvae hatched, but one sample had 704 hatched larvae. During the late summer sampling, an even larger proportion of soil samples were negative (192 of 240 samples). The large variation causes mean values of hatched larvae per soil sample to be misleading and suggest that viable mosquito eggs are not evenly distributed in the soil but occur in clusters. The survival of larvae to adult mosquitoes was around 37% and 2685 mosquitoes could be identified to species. The species distribution of the identified mosquitoes was: *Ae. cinereus* 46%, *Ae. sticticus* 27%, *Ae. rossicus* 14%, *Aedes vexans* 4.4%, *Aedes punctor* 4.1%, *Aedes intrudens* 2.2%, Annulipes complex 1.3%.

**Figure 2:**
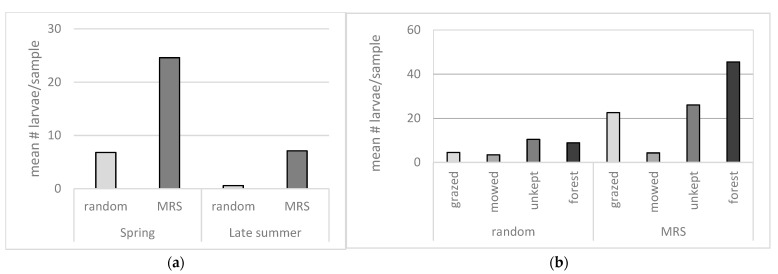
(**a**) Mean number of mosquito larvae hatched per sample in random and moisture retaining structures (MRS) samples, sampled during spring and late summer. (**b**) Mean number of mosquito larvae per sample in the four environmental categories, random, and MRS samples.

The comparison of the environmental categories show that mowed areas yield significantly fewer mosquito larvae than the other categories when both random and MRS samples are compared. In the random samples, only the difference between mowed areas and unkept open areas is significant, while the MRS samples show that mowed areas are significantly different from all other categories, Figure 2b.

**Figure 3:**
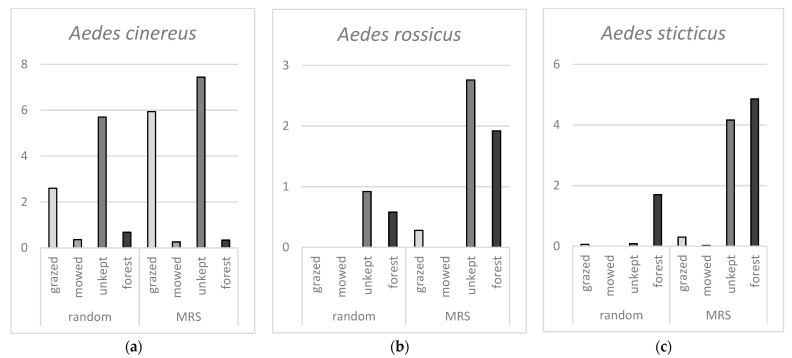
(**a**) Mean number of *Ae. cinereus* larvae hatched per sample in random and MRS samples from different environmental categories. (**b**) Mean number of *Ae. rossicus* larvae hatched per sample in random and MRS samples from different environmental categories. (**c**) Mean number of *Ae. sticticus* larvae hatched per sample in random and MRS samples from different environmental categories.

Results for each of the three most common mosquito species highlight further differences between the different environmental categories, Figure 3. For *Ae. cinereus,* there is no significant difference in number of hatched mosquitoes between random samples and MRS samples. Further, there are significantly more hatched *Ae. cinereus* from grazed and unkept areas compared to mowed and forest areas.

Both *Ae. rossicus* and *Ae. sticticus* hatched significantly more from MRS samples compared to random samples. For *Ae. rossicus,* there was a significant difference between grazed and mowed areas compared to unkept and forest areas where more larvae were hatched from the latter categories. For *Ae. sticticus,* there are significantly more larvae hatched from forest areas compared to the three other categories. Even if the mean values of total hatched larvae for unkept areas and forest areas look similar there are significant differences between these categories. Together, the results show that *Ae. cinereus* occurs mostly in grazed and unkept open grassland areas while *Ae. rossicus* hatch mainly from MRS in unkept open areas and forests, and *Ae. sticticus* show a strong preference for MRS in forests.

To determine whether MRS, such as tussocks, willow shrubs, piles of leaves, logs, and roots are associated with more viable mosquito eggs, targeted samples from such structures were compared to samples taken in random locations from the same area. The results show that significantly more larvae hatched from MRS samples compared to random samples, both during spring and during late summer, Figure 2a.

**Figure 4:**
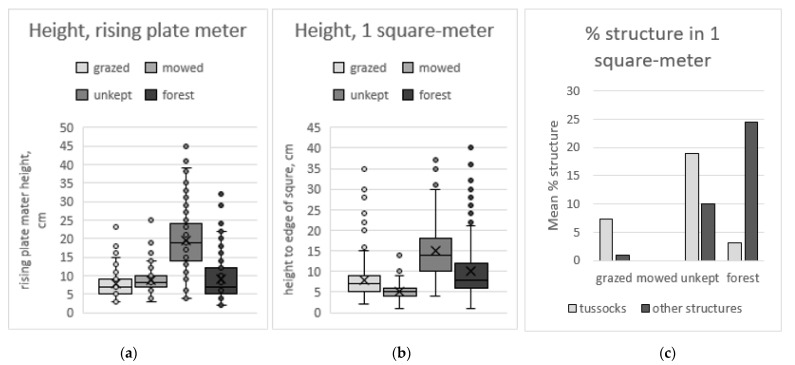
(**a**) Box plot of rising plate meter height measurements from different environmental categories. (**b**) Box plot of 1 square-meter height measurements from different environmental categories. (**c**) Mean percent of 1 square-meter covered by MRS in different environmental categories.

The vegetation height and structural characteristics of the areas were measured using both a rising-plate meter and a 1 square-meter frame, Figure 4. The rising-plate meter shows that the unkept areas have the most vegetation ground cover and tussocks giving the highest values, as well as the larges variation between measurements. All three other categories are more similar, but the variation is higher in the forest category than in grazed and mowed areas. Moreover, the measurements of the distance between the square-meter frame and the ground are larger in unkept open areas reflecting that these areas were covered with high tussocks; this method also indicated that mowed areas were flatter than grazed areas and forest areas.

Comparing the area covered by MRS in each sampling area by assessing how much of the square-meter frame was covered by structures also showed big differences between the categories. Mowed areas had no MRS in the ten sampled frames while grazed areas had 8% of the area covered by structures and unkept areas and forest areas had over 25% of the area covered by structures. When MRS were divided into tussocks and other MRS the difference between grazed areas that mainly had tussocks, unkept areas where a third of the MRS are other kind of structures such as shrubs, and forest areas where there are few tussocks, but much more piles of leaves and branches, logs, and roots that make up MRS.

When soil-samples that were directed towards MRS were collected, a description of each sampled structure was also made. Using this data, it was possible to investigate the type of MRS the different mosquito species were hatched from. In unkept areas, where both tussocks, shrubs of willows, and other MRS were present the difference between the three common mosquito species was evident, where *Ae. cinereus* preferred tussocks, *Ae. rossicus* used both tussocks and other MRS, but *Ae. sticticus* preferred willows, Table 1.

Previous observations suggest [23] that fewer mosquito larvae are hatched from soil samples taken later in the summer compared to earlier in the summer. To address this, samples were taken also in the autumn from 12 of the 20 locations (three from each category) to compare to the samples taken during spring. The comparison shows that significantly more larvae hatched during spring, Figure 2a.

## 4. Discussion

Previous studies have addressed how environmental factors and maintenance such as grazing and mowing affects the mosquito abundance [1,6,7,8,9,19,22,23], with studies also from the Dalälven region. Another report from Dalälven on larval abundance during floods failed to measure any difference [29], but no details on how the study was performed was included. In this study we report significant differences in the amount of hatched mosquito larvae from four environmental categories. The variance in number of larvae hatched from each soil sample is very large in all categories, with a large proportion of samples not producing any larvae whereas other samples hatched several hundred larvae. This indicate that the eggs are clustered in the soil and not randomly spread out which makes mean values of eggs per sample dominated by few outlying samples and less reliable. To compare the categories and sample times nonparametric tests are necessary.

The clustering of eggs as well as our finding that more larvae hatch from MRS samples suggest that female mosquitoes actively choose suitable egg laying positions and that many eggs are clustered in a subset of preferred locations. In container breeding mosquitoes, a mechanism has been reported that eggs laid by one female attracts others to use the same container [30]. Whether a similar mechanism might be active also in floodwater mosquitoes would need to be studied further.

It has previously been reported that not all mosquito eggs hatch when a soil sample is first flooded but that repeated soakings can hatch additional larvae [22,31]. Rydzanicz et al. [22] reported that 75% of eggs were hatched during the first soaking of a sample and that each additional soaking resulted in fewer larvae. Our samples were only soaked once and while we might not count all larvae that could hatch from each sample the differences between the environmental categories should not be affected since all samples were treated in the same way. Silver [24] report that eggs of different species are not equally sensitive to soaking, but that some species hatch earlier than others. This has the potential to affect our results so that we underestimate a species that need longer floods to hatch compared to other species. However, it would not affect the differences between the categories. In comparison with earlier catches of adult mosquitoes in the area [10] there is no species that is missing from our samples that is common in catches of adult mosquitoes.

By allowing the larvae to develop to adult mosquitoes, we were able to identify a large proportion of larvae to species. This allowed comparisons between the three most abundant mosquito species highlighting the differences between them. *Ae. cinereus* dominated in open areas with tussocks, as has been reported for adult *Ae. cinereus* [14,15], while *Ae. sticticus* preferred locations with some foliage cover, such as willow shrubs in otherwise open unkept areas or in forest areas. This finding supports the observation by Gjullin [19] that removal of bushwood lowered abundance of *Ae. sticticus*. One paper compared the number of larvae in mowed and grazed grasslands to unkept grasslands during floods in the Dalälven area [9]. They reported a 70% reduction in larvae, but larvae were not identified to species so the results could represent several species.

This study also addressed whether MRS, such as tussocks, logs, piles of leaves, roots, and shrubs are important for mosquito egg laying. The results show that significantly more mosquito larvae hatch from these structures compared to random soil samples taken in the same area, indicating that these MRS are important. The amount of such structures vary greatly between the four environmental categories, from mowed areas where there are few MRS to bushwood forests where suitable structures are common. While this study did not follow the abundance of mosquitoes before and after interventions, such as grazing or mowing of meadows or clearing of shrubs and bushwood to create open areas, it clearly demonstrates significant differences between these categories suggesting that such interventions might affect mosquito nuisance. It is interesting to note that although MRS cover only 8% of the area in grazed areas, mainly in the form of tussocks, they produce large numbers of mosquitoes, primarily *Ae. cinereus*. This suggests that removing tussocks from grazed areas might reduce the number of mosquitoes hatching from there during flooding significantly. Managed grasslands, managed by mowing and grazing, given that tussocks are removed, seem to be a way to reduce nuisance mosquitoes. However, in a complex area such as Dalälven, the ratio between managed and unkept grasslands, and forests makes it an ineffective method due to the huge areas covered by mosquito producing biotopes compared to the managed areas. Since the flood water forests in the area have very high biodiversity it is not fathomable to manage them in a mosquito reducing way. Our study clearly show that these forested areas are producing the main nuisance mosquito in the area, *Ae. sticticus*. It is striking from these results that studying each mosquito species independently allows a better understanding of the effect environmental factors has on abundance of floodwater mosquitoes, where *Ae. cinereus* and *Ae. sticticus* seem to have very different requirements. Because of these differences in preferred oviposition environment, the differences between the environmental categories are not obvious when total number of larvae are compared.

An earlier report as well as our unpublished observations suggested that seasonal timing might affect the hatching of mosquito larvae from collected soil samples, but no controlled study was performed [23]. The exposure of *Aedes sticticus* eggs to summer temperatures after winter treatment reduced their hatch rate [11]. We compared samples taken from all four environmental categories during spring and late summer and detect a significant difference. During the summer between collections there was no major flooding so the number of eggs in the soil should be roughly equal, even though the survival of the eggs might be affected slightly by the added months. It is plausible that there is some mechanism that could stop eggs from hatching in floods too late in the season when the mosquitoes would not survive to adults and lay new eggs. Further studies would be needed to determine how seasonal timing affects the hatching of floodwater mosquitoes more specifically. However, our results indicate that caution should be taken when comparing samples taken during different seasons and that samples collected in spring give the highest rate of hatching.

## Figures and Tables

**Table 1:** Number of mosquito larvae of the three most common species hatched from MRS in unkept open areas divided by type of structure. The different mosquito species prefer different types of MRS.

	Tussock	Willow	Other Structure	Total
***Aedes cinereus***	245 (65%)	97 (26%)	30 (8%)	372
***Aedes sticticus***	20 (10%)	188 (90%)	0 (0%)	208
***Aedes rossicus***	65 (48%)	62 (45%)	11 (8%)	138

## Data Availability

All mosquito larvae data produced in the project are summarized in Table S1.

## Supplementary Materials

The following are available online at https://www.mdpi.com/2075-4450/12/4/279/s1, Table S1: Samples, raw data, Table S2: sample locations.
